# Supplementary material for: Effects of dietary aromatase inhibitors on masculinization of rosy barb (Pethia conchonius): Evidence from growth, coloration and gonado-physiological changes
Source: PLoS One. 2023 Nov 3;18(11):e0287934. doi: 10.1371/journal.pone.0287934 (PMC10624304; doi:10.1371/journal.pone.0287934)
Supplement: Attachment — Submitted filename: Response to Reviewers 03.06.2023.doc [file pone.0287934.s001.doc]

Respected Editor,

On behalf of the authors, I am very much thankful to the two anonymous reviewers for their insightful comments, constructive criticisms and suggestions which will enhance the quality of the submitted work. We have carefully read and tried our best to accommodate majority of the comments by the reviewers. As mentioned, the response to the specific comments is given below for kind consideration.

**Response to Reviewer #1**

***General comments to the authors:***

This study shows the role of two aromatase inhibitors, 17α-methyltestosterone (MT) and letrozole, in enhancing reproduction, growth, coloration and physiological profile changes. I tried to connect the work to previous reports and noticed that many work on MT have been reported, whereas the second AI, letrozole, is a new entrant in fish studies. The work's novelty, in which gel-based diets are introduced as a delivery vehicle, is applauded. Earlier studies on
physiological consequences and coloration were also not well defined, as attempted in this work. However, there are still some points need to improve as below.

**Specific comments to authors and response to the queries**

| **No.** | **Comment** | **Response** |
| --- | --- | --- |
| 1. | Title: "Effects of dietary aromatase inhibitors on Rosy Barb, Pethia conchonius, masculinization: evidence from growth, coloration, and gonado-physiological changes." | Modified the title as suggested. |
| 2. | Line 34, "for 90 days," can be removed. | Removed as suggested. |
| 3. | Lines 39-42: Rewrite the sentence. | Rewritten as suggested. |
| 4. | Introduction:  Aromatase inhibitors' second mention in this section and elsewhere may be abbreviated. | Changed as suggested. |
| 5. | Lines 83-93 may include recent reports on the use of gels in fish feed. | No specific kinds of literature are found on the use of gels in fish feed. |
| 6. | Materials and procedures:  Known units can be written in abbreviated form. Hours, for example, can be written as hr. | Modified as suggested. |
| 7. | Line 207: Include a reference | Magnifications 10X and 40X are used. |
| 8. | Section 2.9: Magnification is required. |  |
| 9. | Statistical evaluation:  Were the results normalized? Mention the methods that were used. | There is both One-way & Two-way ANOVA used. |
| 10. | Results:  P values can be written in a consistent manner. It has been noticed that it is written as "P" and "p" in many places. | Modified as suggested. (P≤0.05). |
| 11. | Discussion:  The use of words like "mild" is highly unscientific. | Replaced these words as suggested. |
| 12. | Section 4.4 is quite lengthy | Sentence is modified as suggested. |
| 13. | The same is true for Section 4.5. | Sentence is modified as suggested. |
| 14. | The authors must also explain why the gel-based diet was superior to previous studies. | The gel-based feed is an eco-friendly higher water stable and minimal leaching nutrients for a clean environment. |
| 15. | This serves as the foundation and highlights the work's uniqueness | Sex reversal in ornamental fish rosy barb male is brighter red color than female, so this work is filled the sexual attributes for the better market demand. |
| 16. | The conclusion could be improved. |  |
| 17. | Present what you have, the gaps that have been identified, and how future research can be used to fill them. | The gel-based feed is used as AI delivery for fish sex reversal, more doses and feeding trial period required, future research needs to more dose of LET/MT for complete masculinization. |

**Response to Reviewer #2**

**Specific comments**

| **No.** | **Comment** | **Response** |
| --- | --- | --- |
| 1. | Research topic is highly recommended for publishing with standard english writing. The whole manuscript is to be rechecked for grammatical errors. | Revised throughout as suggested. |
| 2. | Growth and coloration of fishes are very much influenced by tank dimensions and light availability. | 12:12 Dark:Light. |
| 3. | Authors are advised to mention the type of tanks used during the experiments and the light arrangement in the experimental aquarium. | Aquarium tank, No specific light arrangement. |
| 4. | Explanations may be incorporated for the selection of particular doses of letrozole (LET) during the current experiment. | Findings from the prevoius studied. |
| 5. | The nutritional composition of feed used during the current experiment needs to be incorporated to substantiate the unique proposal of gel feed for fishes. | Given in the table number 1. |
| 6. | The following paragraphs need to be revised for grammar corrections and for better clarity about the topics | Changed in all places as suggested. |
| 7. | 253- 254- How minimum mortality can be 82.22 %? | Changed to “mortality 6.66%”. |
| 8. | 229 to 234 – Grammatical corrections with clear descriptions are needed. | Corrected as suggested. |
| 9. | 281-291 - Rewrite the sentence | Rewritten as suggested. |
| 10. | 297-301- Grammatical corrections are required. | Corrected as suggested. |
| 11. | The discussion part is poorly written and needs revision. The following sentences are lacking in meaning. • 310-313 • 365-374 • 379-428 | Revised all the sentences throughout as suggested. |
| 12. | Conclusion to be amended for impressive illustrations of experiment. | We had done as the reviewer suggested. |

We look forward for further comments/clarification if any so as to make the MS a valuable and perfect presentation to the readers.

Yours faithfully,

(Soibam Khogen Singh)
